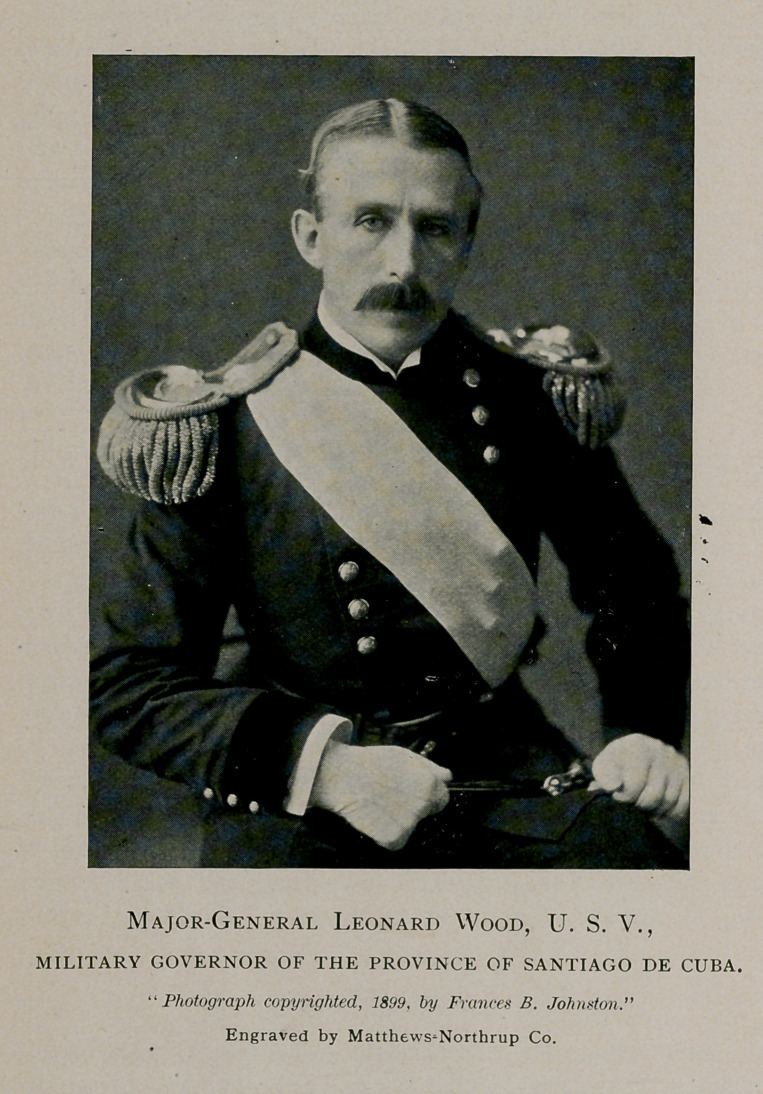# General Leonard Wood

**Published:** 1899-04

**Authors:** 


MAJOR-GENERAL LEONARD WOOD, U. S. V.,

MILITARY GOVERNOR OF THE PROVINCE OF SANTIAGO DE CUBA.

*"Photograph copyrighted, 1899, by Frances B. Johnston."*

Engraved by Matthews-Northrup Co.

### BUFFALO MEDICAL JOURNAL.

**Monthly Review of Medicine and Surgery.**

*EDITORS:*

THOMAS LOTHROP, M. D. - WM. WARREN POTTER, M. D.

All communications, whether of a literary or business nature, should be addressed to the managing editor · 284 FRANKLIN STREET, BUFFALO, N. Y.

VOL. XXXVIII. APRIL, 1899. NO. 9.

### GENERAL LEONARD WOOD.

MAJOR-GENERAL LEONARD WOOD, the Military Governor of the Province of Santiago de Cuba, is one of the strongest and most unique characters that our war with Spain has developed. We have made reference to General Wood before in these columns and take pleasure in presenting to our readers in this issue his picture, made from one of his latest photographs. It was taken by Miss Frances B. Johnston during General Wood's recent visit to this country and is the first picture taken of him in the full dress uniform of a major-general.

General Wood's successful career in Cuba is of special interest to doctors because he himself is a physician—the only physician by profession to attain the grade of general officer during our war with Spain. When the war began he was an assistant surgeon in the U. S. Army, with the rank of Captain, stationed at Washington, and by assignment was attending army officers and their families. Governor Roosevelt was then assistant secretary of the navy and an intimate acquaintance, that began when the two were out on the plains, continued to grow as they frequently met during their ante bellum sojourn at the capital. Finally, when war came, these two strong men were authorised to raise a regiment of cavalry at large, the doctor to be colonel and the secretary to be lieutenant-colonel. This was done, and when it was gathered at San Antonio, the place of rendezvous, it was found to contain not only men of all nations, but from every walk of life, from college professor to cow puncher, and it was appropriately popularly named "the rough riders."

By dint of greatest energy on the part of the field officers, it was equipped and reached Tampa in season for two battalions to be designated for service in Cuba. These formed a pait of Shafter's expedition and succeeded in landing at Daiquiri, June 22, 1898, as part of the cavalry division under command of Major-General Joseph Wheeler. Pushing on toward Santiago the division found the enemy at La Quasina, where there was a stiff engagement June 26th, during which Captain Capron and Sergt. Hamilton Fish among others were killed. For his skill and valor at this fight Colonel Wood was promoted to Brigadier-General, and for similar reasons Roosevelt became Colonel of the rough riders. The two battalions further distinguished themselves at San Juan, and after the surrender General Wood was appointed Military Governor of Santiago, succeeding General McKibbon, who became ill.

General Wood took office July 20, 1898, and still continues to perform its functions. At that time Santiago was probably the filthiest city in the world, its inhabitants were sick and starving and 200 of its people were dying every day. The difficulties to be overcome were such as might quail the stoutest heart. But General Wood was not the man to be dismayed or to shrink from duty because of its magnitude. Here was an opportunity for a man of inexhaustible power of resource, and a physical stamina that no work could tire to display his skill, patience, tact, judgment, statesmanship and courage. A physician who was a sanitarian, a soldier who was a disciplinarian, and a statesman who was a financier and diplomat — all these were needed in the person who should attempt to administer the affairs of Santiago. It appears in the light of after events that the hour and the opportunity and the man met, when General Wood was assigned to his herculean task.

By education and practice he was first a physician, then as emergencies presented on the plains he demonstrated his soldierly qualities and he was often placed in charge of expeditions against the Indians. He had not been very long in control at Santiago before he exhibited the highest skill in statecraft, bringing order out of chaos, administering the law in a firm but just manner and harmonising all interests with a skill and firmness that has challenged the admiration of the civilised world.

In Santiago he has been at once governor, judge, financial officer, school superintendent, chief of police, health commissioner, water commissioner, street commissioner, in short, the whole machinery of civil and military government has been centered in this one man, and he has been equal to his trust in all respects.

Mr. Henry Harrison Lewis, who paid a visit to Santiago last fall, has published an article on Americanising a Cuban city in McClure's magazine for March. It should be read by everybody and especially the young, who ought to be stimulated thereby to the best work of which they are capable. We cannot forego the temptation to quote the conclusion of Mr. Lewis's paper, which reads as follows :

At the time I concluded my visit of observation, there had been just four months of American rule in Santiago de Cuba. Those four months had effected :

The rescue of the population from starvation to a fair satisfaction of all their daily necessities.

The conversion of one of the foulest cities on earth to one of the cleanest.

The reduction of an average daily death-rate of 200 down to ten.

A considerable progress in a scheme of street and road improvement that will add immensely to the convenience and beauty of the city.

A radical reform in the custom-house service, resulting in increased revenues.

A reduction in the municipal expenses.

The correction of numerous abuses in the management of jails and hospitals and in the care of the inmates.

The liberation of many prisoners held on trivial or no charges.

The reformation of the courts and a strict maintenance of law and order.

The freedom of the press.

A restoration of business confidence, and a recovery of trade and industry from utter stagnation to healthy activity.

This unparalleled regeneration had been wrought, not by a host of men native to the locality, exercising offices long established and enjoying a traditional prestige, but by an American brigadier-general of volunteers, a stranger to the place and the people, embarked in the work on a moment's notice, and having for his immediate aides only a few fellow army officers, some of whom had been out of West Point less than two years, and all of whom were as new to the situation as himself. It was the *tour de force* of a man of genius ; for in the harder, more fundamental, of the tasks that confronted him here, General Leonard Wood had had no previous experience.

It has fallen to the lot of but few men of thirty-eight years to demonstrate such rare qualities of head and heart as has the young physician, or to attain the universal respect of their fellow-countrymen.

## Figures and Tables

**Figure f1:**